# A randomized controlled trial of green tea catechins in protection against ultraviolet radiation–induced cutaneous inflammation^1^,^2^

**DOI:** 10.3945/ajcn.115.107995

**Published:** 2015-07-15

**Authors:** Mark D Farrar, Anna Nicolaou, Kayleigh A Clarke, Sarah Mason, Karen A Massey, Tristan P Dew, Rachel EB Watson, Gary Williamson, Lesley E Rhodes

**Affiliations:** 3 Centre for Dermatology, Institute of Inflammation and Repair, University of Manchester, Manchester Academic Health Science Centre, Salford Royal NHS Foundation Trust, Manchester, United Kingdom;; 4 Manchester Pharmacy School, Faculty of Medical and Human Sciences, University of Manchester, Manchester, United Kingdom;; 5 School of Food Science and Nutrition, University of Leeds, Leeds, United Kingdom; and; 6 Bradford School of Pharmacy, Faculty of Life Sciences, University of Bradford, Bradford, United Kingdom

**Keywords:** green tea catechins, human skin, inflammation, photoprotection, ultraviolet radiation

## Abstract

**Background:** Safe systemic protection from the health hazards of ultraviolet radiation (UVR) in sunlight is desirable. Green tea is consumed globally and is reported to have anti-inflammatory properties, which may be mediated through the impact on cyclooxygenase and lipoxygenase pathways. Recent data suggest that green tea catechins (GTCs) reduce acute UVR effects, but human trials examining their photoprotective potential are scarce.

**Objective:** We performed a double-blind, randomized, placebo-controlled trial to examine whether GTCs protect against clinical, histologic, and biochemical indicators of UVR-induced inflammation.

**Design:** Healthy adults (aged 18–65 y, phototypes I–II) were randomly allocated to 1350 mg encapsulated green tea extract (540 mg GTC) with 50 mg vitamin C or placebo twice daily for 3 mo. Impact on skin erythema, dermal leukocytic infiltration, and concentrations of proinflammatory eicosanoids was assessed after solar-simulated UVR challenge, and subject compliance was determined through assay of urinary GTC metabolite epigallocatechin glucuronide.

**Results:** Volunteers were assigned to the active (*n* = 25) or the placebo (*n* = 25) group. After supplementation, median (IQR) sunburn threshold (minimal erythema dose) was 28 (20–28) and 20 (20–28) mJ/cm^2^ in the active and placebo groups, respectively (nonsignificant), with no difference in AUC analysis for measured erythema index after a geometric series of 10 UVR doses. Skin immunohistochemistry showed increased neutrophil and CD3^+^ T-lymphocyte numbers post-UVR in both groups (*P* < 0.01) with no statistically significant differences between groups after supplementation. Cyclooxygenase and lipoxygenase metabolites prostaglandin E_2_ (vasodilator) and 12-hydroxyeicosatetraenoicacid (chemoattractant), respectively, increased after UVR (*P* < 0.05), with no differences between supplementation groups.

**Conclusion:** Oral GTC (1080 mg/d) with vitamin C over 3 mo did not significantly reduce skin erythema, leukocyte infiltration, or eicosanoid response to UVR inflammatory challenge. This trial was registered at clinicaltrials.gov as NCT01032031.

## INTRODUCTION

The skin has important roles in the body’s defense against harmful stimuli. Ultraviolet radiation (UVR)^7^ in sunlight is a key environmental trigger of acute adverse events, including dose-related inflammation (sunburn), photosensitivity, immunosuppression, and DNA damage, with repeated exposure leading to chronic photodamage and photocarcinogenesis. Topical sunscreens can protect by absorbing and scattering UVR but have drawbacks, including generally infrequent application outside of holiday times and inadequate application methods, with uneven spread and use at surface density considerably lower than the manufacturers’ sunscreen testing conditions (1, 2). A dietary approach to photoprotection could provide a continuous adjunctive measure, with population-level impact (3).

The acute response of the skin to UVR is orchestrated by cells within the epidermal and dermal layers that release mediators producing acute inflammation, which presents clinically as erythema and histologically as a dermal leukocytic infiltrate (4, 5). UVR-induced oxidative stress stimulates expression and activity of cytosolic phospholipase A_2_, leading to increased production of free arachidonic acid, which can be metabolized by cyclooxygenases and lipoxygenases to a wide range of eicosanoids (6, 7), many of which play roles in inflammation, notably the cyclooxygenase-2 product prostaglandin (PG) E_2_, a major mediator of UVR-induced erythema (8, 9), and the 12-lipoxygenase–derived 12-hydroxyeicosatetraenoicacid (HETE), a potent leukocyte chemoattractant (9–11) in the sunburn response.

There is considerable interest in the potential benefits to health of green tea, which is widely consumed in many parts of the world. In addition to a broad literature relating to their chemopreventive activities (12, 13), green tea catechins (GTCs) have anti-inflammatory properties (14, 15) and are part of the polyphenol group, which can reduce the expression and activity of cyclooxygenase-2 and several lipoxygenase isoforms (16–19). The green tea polyphenol (−)-epigallocatechin-3-gallate (EGCG) has been reported to reduce cyclooxygenase-2 expression, in addition to UVB-mediated activation of nuclear transcription factor κB and neutrophil migration in vitro (20–23), and oral GTCs were protective against UVR-induced skin inflammation as well as carcinogenesis in hairless mice (24). In humans, topical application of GTCs before UVR reduced cutaneous erythema, leukocyte infiltration, and DNA damage induced by UVR (25), whereas oral consumption was reported to increase the clinical erythema threshold to UVR (26). In a before-after pilot study, 12-wk oral GTCs led to incorporation of GTC metabolites into skin and modest protection against UVR-induced erythema, alongside reduced 12-HETE production (27).

The above findings indicated the requirement for a double-blind, randomized controlled trial of adequate sample size to examine in more depth the photoprotective potential of oral GTCs against acute UVR insult. We have conducted such a study of 3-mo oral GTCs with low-dose vitamin C in healthy humans, evaluating its ability to protect against UVR-induced inflammation by *1*) visual and objective assessment of clinical erythema after a range of UVR doses, *2*) histologic assessment of skin leukocytic infiltrate, and *3*) quantification of the major proinflammatory cyclooxygenase- and lipoxygenase-derived mediators upregulated in sunburn, PGE_2_, and 12-HETE.

## METHODS

### Study design

Fifty healthy white adults (aged 18–65 y), male and female, Fitzpatrick sun-reactive skin types I–II, were recruited by open advertisement. Exclusion criteria were history of skin cancer or photosensitivity disorder, sunbed use/sunbathing in the 3 mo before the study, taking photoactive medication or nutritional supplements, consuming >2 cups of tea/d, or currently pregnant or breastfeeding. Ethical approval was obtained from the North Manchester Research Ethics Committee (reference 08/H1006/79). Written informed consent was obtained from the participants and the study adhered to Declaration of Helsinki principles. The study was conducted in the Photobiology Unit, Dermatology Centre, Salford Royal Hospital, Manchester, United Kingdom, between November 2010 and August 2011. Subjects were randomly assigned to receive green tea extract plus vitamin C or placebo maltodextrin (1:1; block randomization with random block size between 4 and 8; StatsDirect v2.7.8, StatsDirect Ltd.). Containers containing active and placebo supplement were sequentially numbered. Subjects and investigators were blinded to the intervention, and the randomization code was held securely until completion of the study.

Green tea supplements were gelatin capsules each containing 450 mg green tea extract (180 mg GTC) (27) and a further set of capsules each containing 25 mg vitamin C. Actively supplemented subjects took 3 green tea and 2 vitamin C capsules twice daily (with breakfast and evening meal; total daily dose 1080 mg GTC, 100 mg vitamin C; **Table 1**) for 12 wk. Low-dose vitamin C stabilizes the green tea extract in the gut lumen and has been shown to have no impact itself on UVR erythema (28, 29). Placebo subjects took supplements comprising maltodextrin in gelatin capsules of identical appearance to those containing green tea and vitamin C. The primary outcome was change in the minimal erythema dose (MED) of UVR at 12 wk. Secondary outcomes were change in UVR-induced neutrophils and CD3^+^ T lymphocytes in skin biopsy sections, as well as PGE_2_ and 12-HETE in suction blister fluid at 12 wk. All supplements were provided by Nestec Ltd. and packaged in bottles with identical appearance by Laboratoire LPH. Compliance was assessed by counting residual capsules in dispensed containers returned by volunteers and measurement of the green tea urinary marker epigallocatechin glucuronide as described below.

**TABLE 1:** Total daily amount of green tea extract constituents consumed by active group subjects

Green tea constituent	Amount, mg
Gallic acid	1.8
Catechin	12.6
Epicatechin	75.0
Gallocatechin	74.4
Epigallocatechin	295.8
Catechin gallate	1.8
Epicatechin gallate	156.0
Gallocatechin gallate	28.0
Epigallocatechin gallate	435.6

### UVR exposure and assessment of erythema response

Exposures were performed by using a solar simulator with emission of UVB and UVA mimicking that of sunlight (emission 290–400 nm; 5% UVB, 95% UVA; Newport Spectra-Physics Ltd). Irradiance was measured 10 cm from the source before each irradiation by using a radiometer (model IL 730A; International Light) calibrated for use with the light source to ensure consistency of doses applied.

The MED of UVR (i.e., the sunburn threshold) for each subject was assessed at baseline and postsupplementation, after application of a geometric series of 10 doses of solar-simulated UVR (erythemally weighted, 7–80 mJ/cm^2^) to upper buttock skin (1-cm-diameter circular sites). Irradiated sites were examined visually after 24 h, with the MED defined as the lowest dose producing visually discernible erythema.

Erythema intensity at each of the 10 UVR-exposed sites was quantified by using a reflectance instrument (Diastron). Readings were taken in triplicate from each exposed site and from adjacent unexposed skin and erythema expressed as the difference between these readings (ΔE). Dose-response modeling was performed by using a dedicated data analysis package (Regional Medical Physics Department, Gateshead & Tyneside Health Authority) to calculate each subject’s D_30_, the UVR dose producing a ΔE of 30 arbitrary units, which is a threshold value approximating an individual’s visual MED.

### Skin biopsy and suction blister fluid sampling

At 24 h before skin tissue and blister fluid sampling, doses of UVR of 3× the individual’s presupplementation MED were given to sites on one buttock to provoke an inflammatory response sufficient to significantly elevate cutaneous eicosanoid concentrations (9). UVR-exposed and UVR-protected areas of upper buttock skin were sampled at baseline and postsupplementation; UVR exposures were limited to one buttock, and the other buttock provided the unexposed skin and blister fluid samples. Skin punch biopsy specimens (5 mm diameter) were taken after intradermal injection of lignocaine as described (9), snap frozen, and stored at −80°C. Suction blisters were raised by using suctions cups with a central aperture diameter of 1 cm and vacuum of 250 mm Hg as described previously (9). Skin blister fluid was aspirated with a 23-gauge needle, snap frozen in liquid nitrogen, and stored at −80°C until analysis.

### Immunohistochemical staining and assessment

Monoclonal anti–neutrophil elastase (clone NP57) and polyclonal anti-CD3 antibodies were obtained from Dako UK Ltd. Briefly, frozen biopsy specimens were sectioned (5 μm) and endogenous peroxidase activity blocked by incubation in 0.6% (vol:vol) hydrogen peroxide in methanol. Sections were incubated overnight at 4°C with primary antibody and then visualized by using the ImmPRESS system or Vectastain Elite ABC kit (Vector Laboratories Ltd.) for neutrophil elastase and CD3, respectively. All sections were blinded and randomized before assessment. For each biopsy sample, 3 high-power fields (hpfs) were assessed microscopically from each of 3 biopsy sections. Cell number per hpf was determined for neutrophils and CD3^+^ T lymphocytes.

### Analysis of skin blister fluid

Eicosanoids in skin blister fluid were quantified by liquid chromatography coupled to electrospray ionization tandem mass spectrometry as described previously (30–32). Briefly, skin fluid samples (typically 50–200 μL) were diluted with methanol-water (15% w/w) up to 3 mL. Internal standards (40 ng PGB_2_-*d*4 and 80 ng 12-HETE-*d*8; Cayman Chemicals) were then added and resultant solutions acidified to pH 3.0, followed by solid-phase extraction (C18-E cartridges; Phenomenex) to reduce matrix effects and semi-purify the lipid mediators. Eicosanoids were analyzed on a C18 column (Luna 5 μm; Phenomenex) by using a Waters Alliance 2695 HPLC pump coupled to a triple-quadrupole mass spectrometer equipped with an electrospray ionization probe (Quattro Ultima; Waters). Instrument control and data acquisition were performed by using MassLynx 4.0 software (Waters). Multiple-reaction monitoring transitions used were as follows: PGE_2_, *m/z* 351 > 271; PGE_1_, *m/z* 353 > 317; PGE_3_, *m/z* 349 > 269; PGJ_2_, *m/z* 333 > 271; PGD_1_, *m/z* 353 > 317; PGD_2_, *m/z* 351 > 271; PGF_2α_, *m/z* 353 > 193; 13,14-dihydro-15-keto PGE_2_, *m/z* 351 > 333; 13,14-dihydro-15-keto-PGE_1_, *m/z* 353 > 335; 12-HETE, *m/z* 319 > 179; 11-HETE, *m/z* 319 > 167; 15-HETE, *m/z* 319 > 175; 15-hydroxyeicosatrienoic acid, *m/z* 321 > 221; 9-hydroxyoctadecadienoic acid, *m/z* 295 > 171; and 13-hydroxyoctadecadienoic acid, *m/z* 295 > 195.

### Urinary analysis of GTC metabolites

The urinary GTC metabolite epigallocatechin glucuronide was assayed by liquid chromatography–tandem mass spectrometry as previously described (33) to establish compliance with supplementation. Volunteers provided 24-h urine samples before supplementation and after 1 d, 6 wk, and 12 wk of supplementation.

### Statistical analyses

Based on previous oral flavonoid photoprotection studies (34, 35), a sample size of at least 22 subjects per group was estimated to be required to detect a 25% difference in the MED and UVR erythema dose-response between groups, at a 5% significance level with 90% power. Differences in UVR-induced inflammatory and eicosanoid responses between active and placebo groups postsupplementation were compared by ANCOVA of ln-transformed data with baseline data as the covariate. Intent to treat was the primary analysis for comparisons of outcomes between treatment groups with multiple imputation of missing data (*n* = 50). A per-protocol analysis was also performed (*n* = 45) to assess effectiveness of the supplementation. Wilcoxon’s Signed Rank test was used to compare unexposed and UVR-exposed skin within groups. Analyses were performed by using SPSS 20 (SPSS, Inc.). Erythemal dose-response curves were analyzed by using GraphPad Prism (v5.01; GraphPad Software). Statistical significance was accepted at *P* < 0.05.

## RESULTS

### Study subjects and compliance

**FIGURE 1:**
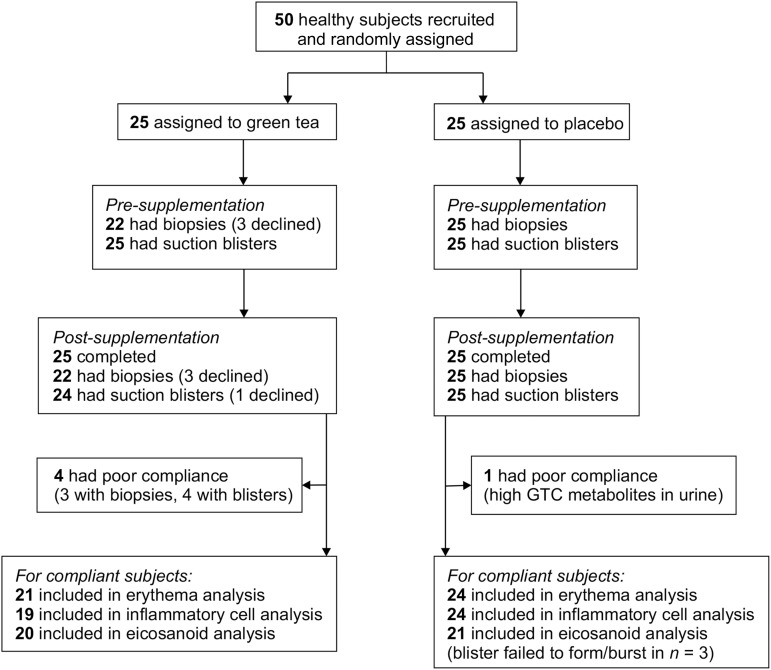
Number of participants randomly allocated and included in analyses. Fifty volunteers enrolled in the study between November 2010 and May 2011. The green tea group took 1350 mg green tea extract (containing 540 mg GTC) with 50 mg vitamin C twice daily. The placebo group took maltodextrin twice daily. GTC, green tea catechin.

Of the 50 subjects recruited to the study, 25 were randomly allocated to active supplementation and 25 to placebo. Baseline characteristics of subjects are shown in **Table 2**. Four subjects in the active group were noncompliant; 2 had a low concentration of epigallocatechin glucuronide in urine at week 12, and 2 had missing urine samples for day 1 and week 12. One subject in the placebo group was noncompliant due to a high concentration of green tea metabolites in urine at day 1 (**Figure 1**). The BMI (in kg/m^2^) of the participants was unchanged throughout the study, with a mean ± SD of 27.7 ± 5.0 and 25.5 ± 4.0 in the active group and 27.9 ± 5.4 and 25.3 ± 3.8 in the placebo group at the 6- and 12-wk points, respectively. The supplement was well tolerated; 6 subjects (5 in the active group and 1 in the placebo group) reported occasional mild nausea after ingestion.

**TABLE 2:** Baseline characteristics of subjects

Characteristic	Active (*n* = 25)	Placebo (*n* = 25)
Age, y	36 ± 13.6^1^	35 ± 12.3
Sex, M/F, *n*	6/19	7/18
BMI, kg/m^2^	27.9 ± 5.4	25.5 ± 3.8
Skin type, I/II, *n*	3/22	1/24
MED,^2^ mJ/cm^2^	28 (12–48)^3^	28 (7–48)

1 Mean ± SD (all such values).

2 MED, minimal erythema dose.

3 Median; range in parentheses (all such values).

### Urinary excretion of epigallocatechin glucuronide

**FIGURE 2:**
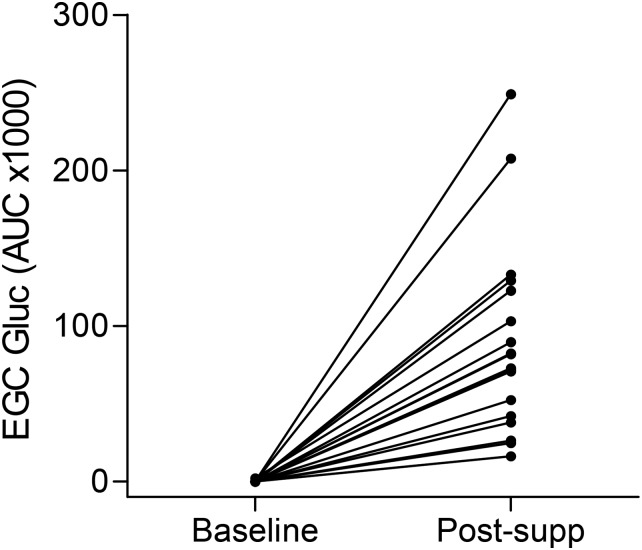
Excretion of EGC glucuronide in urine. Individual urinary concentrations of EGC glucuronide are shown for compliant participants in the active group (*n* = 18) expressed as area under curve per total urine excreted over 24 h. EGC, epigallocatechin; Gluc, glucuronide; post-supp, after supplementation.

Urinary excretion of the green tea metabolite epigallocatechin glucuronide by compliant participants in the active group is shown in **Figure 2**. At baseline, 1 subject in the active group had a low concentration of urinary epigallocatechin glucuronide (AUC = 2094), whereas for all other subjects, the metabolite was not detected. At 6 wk, mean concentration (AUC) in the active group was 75,381 rising to 89,626 at 12 wk. In the placebo group, epigallocatechin glucuronide was undetectable in all subjects pre- and postsupplementation.

### UVR erythema responses

**FIGURE 3:**
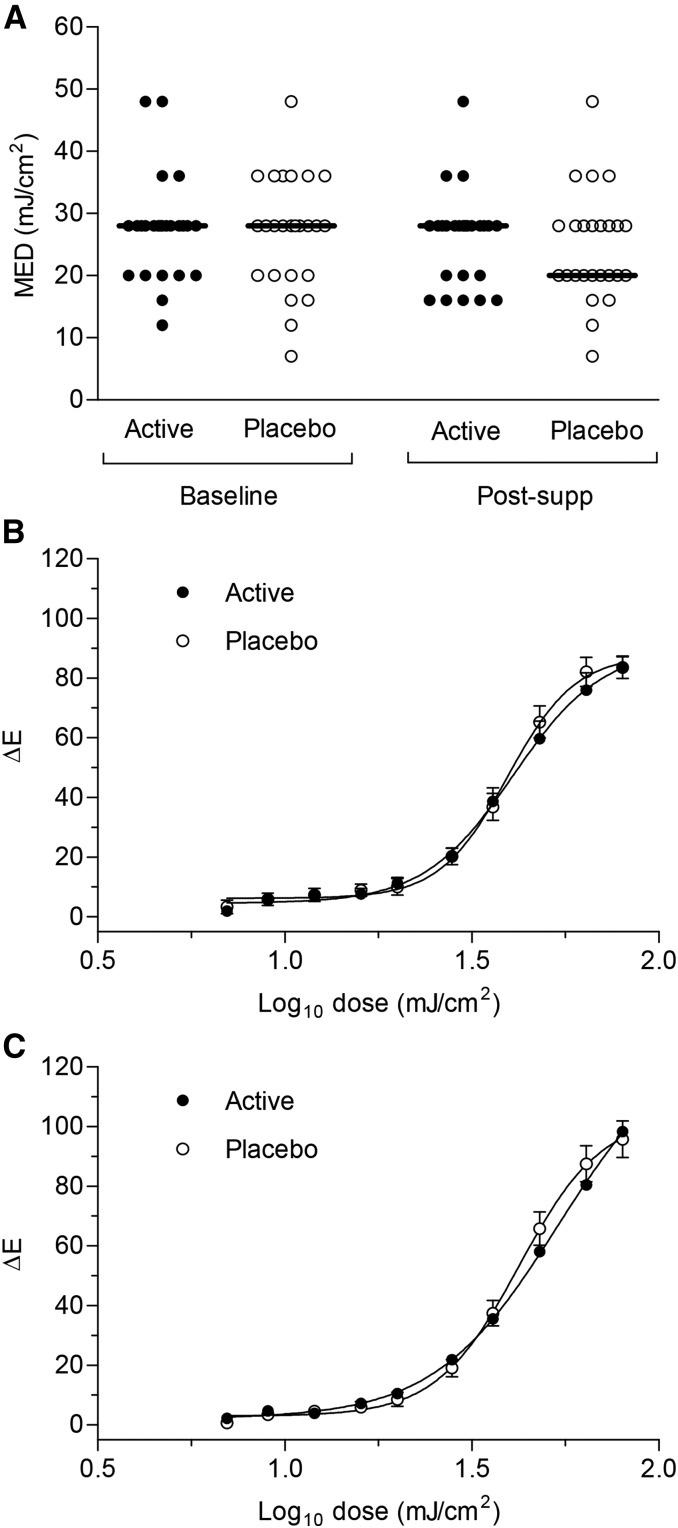
UVR erythema responses at baseline and after 12 wk supplementation. (A) Minimal erythema dose of solar-simulated UVR at baseline and after supplementation with active or placebo treatment. Individual data are shown with horizontal bars representing group medians. Mean ± SEM ΔE is shown for each UVR dose at baseline (B) and post-supp (C). The UVR erythema dose-response was plotted by using a 4-parameter logistic curve (*n* = 25 active; *n* = 24 placebo). MED, minimal erythema dose; post-supp, after supplementation; UVR, ultraviolet radiation; ΔE, difference in erythema intensity between the UVR-exposed site and adjacent unexposed skin.

Postsupplementation, there was no statistically significant difference in visual threshold erythema, that is, in the median MED between the active (28 mJ/cm^2^; range: 16–48) and placebo (20 mJ/cm^2^; range: 7–48) groups (*P* = 0.47; **Figure 3**A). AUC analysis for the measured erythema induced by a series of 10 UVR doses also revealed no statistically significant difference in the UVR erythema dose-response between the 2 groups at baseline (mean 3423 vs. 3573, respectively; *P* = 0.67; Figure 3B) or postsupplementation (mean 3555 vs. 3839; *P* = 0.44; Figure 3C). Within-group analyses showed there was no statistically significant difference in median MED postsupplementation compared with baseline for the active group (both 28 mJ/cm^2^; *P* = 0.17) or placebo group (20 and 28 mJ/cm^2^, respectively; *P* = 0.12).

### Dermal leukocytic infiltration

**FIGURE 4:**
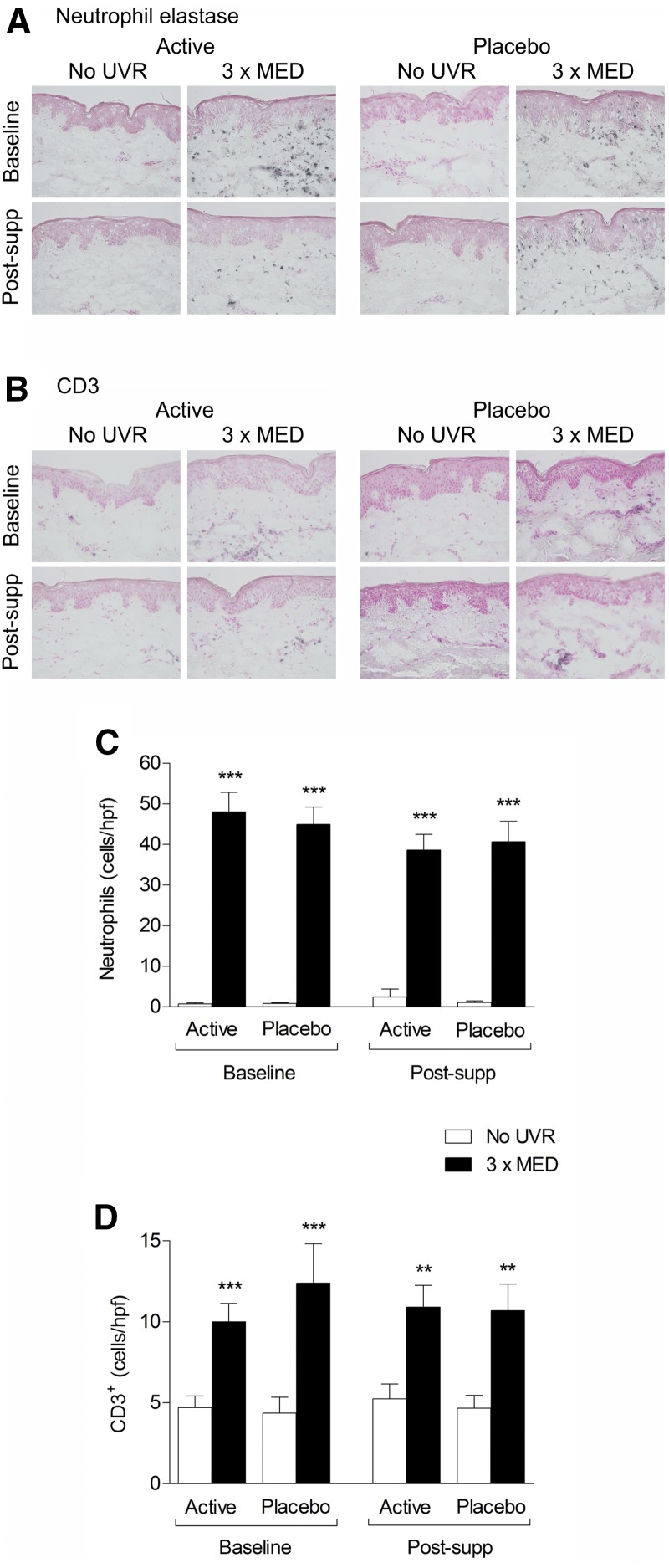
UVR-induced neutrophil and T-cell infiltration at baseline and after 12 wk of supplementation. Representative immunohistochemistry of (A) neutrophils and (B) CD3^+^ T cells in ultraviolet-exposed and unexposed skin at baseline and post-supp. Mean + SEM number of (C) neutrophils and (D) CD3^+^ T cells per high-power field (*n* = 22 active; *n* = 25 placebo). **P < 0.01 and ***P < 0.001, compared with unirradiated skin (Wilcoxon’s Signed Rank test). hpf, high-power field; MED, minimal erythema dose; post-supp, after supplementation; UVR, ultraviolet radiation.

Immunohistochemical assessment of skin sections demonstrated that the number of neutrophils and CD3^+^ T lymphocytes in skin irradiated with 3× MED UVR was statistically significantly higher than in unirradiated skin in both groups, both at baseline and postsupplementation (all *P* < 0.01; **Figure 4**). There was no statistically significant difference in neutrophil numbers in UVR-irradiated skin between groups postsupplementation (*P* = 0.85). Neutrophil numbers in UVR-exposed skin were mean ± SEM of 46.2 ± 5.0 cells/hpf at baseline and 38.4 ± 4.1 cells/hpf postsupplementation (*P* = 0.06) in the active group and 44.2 ± 4.3 and 40.8 ± 5.2 cells/hpf, respectively (*P* = 0.37), in the placebo group. There was little change in the number of CD3^+^ T cells in UVR-exposed skin from subjects in the active group postsupplementation compared with baseline, with no statistically significant difference between active and placebo (*P* = 0.62; Figure 4). Similarly, no statistically significant difference between treatment groups was found after multiple imputation or per-protocol analyses.

### Cutaneous production of eicosanoids

**FIGURE 5:**
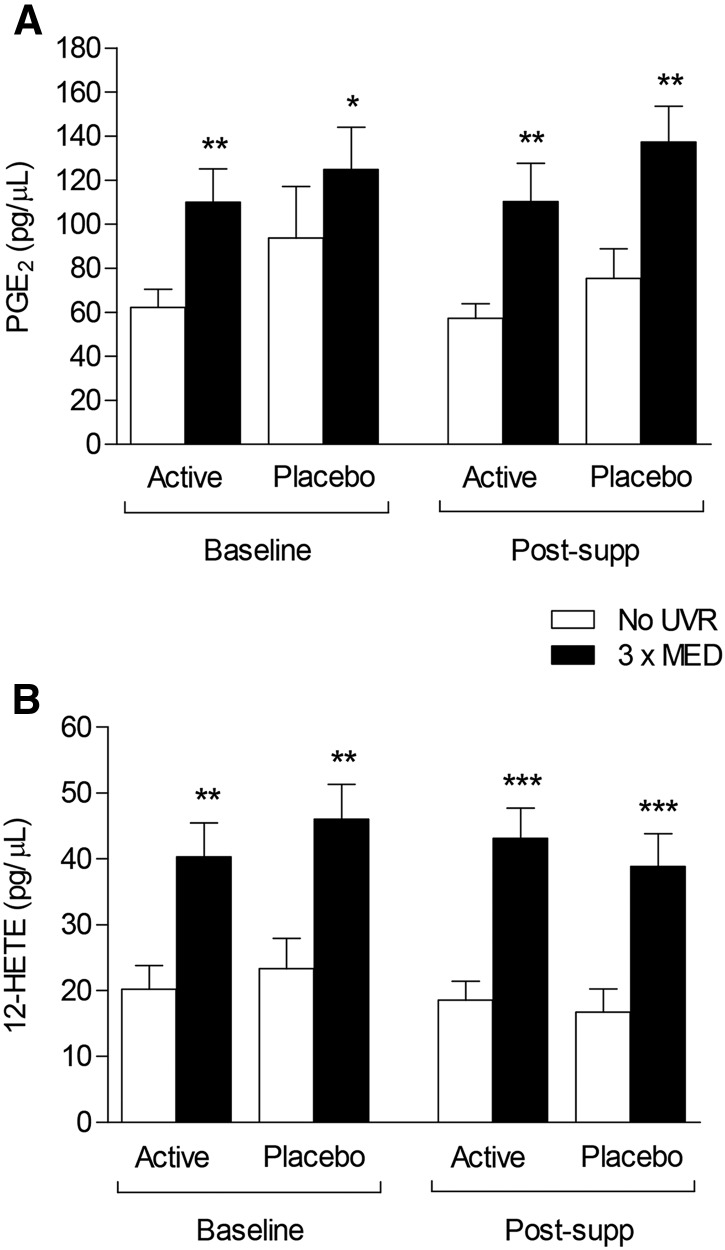
UVR-induced PGE_2_ and 12-HETE production at baseline and after 12 wk of supplementation. Mean + SEM concentration of (A) PGE_2_ and (B) 12-HETE in suction blister fluid from UVR-exposed and unexposed skin at baseline and post-supp (*n* = 20 active; *n* = 21 placebo). * *P* < 0.05, ** *P* < 0.01, and *** *P* < 0.001 compared with unirradiated skin (Wilcoxon’s Signed Rank test). MED, minimal erythema dose; PGE_2_, prostaglandin E_2_; post-supp, after supplementation; UVR, ultraviolet radiation; 12-HETE, 12-hydroxyeicosatetraenoic acid.

We examined 15 eicosanoids, including those of principal interest (i.e., PGE_2_ and 12-HETE). Concentrations of both PGE_2_ and 12-HETE in suction blister fluid from skin irradiated with 3× MED UVR were significantly higher (mean increase post-UVR of 161% for PGE_2_ and 233% for 12-HETE) than in unirradiated skin in both groups, at baseline and postsupplementation (all *P* < 0.05; **Figure 5**). Postsupplementation, the mean ± SEM concentration of PGE_2_ in blister fluid from UVR-exposed skin of subjects in the active group (110.3 ± 17.3 pg/μL) was not significantly different from placebo (137.3 ± 16.3 pg/μL; *P* = 0.35; Figure 5A). Similarly, there was no significant difference in concentration of 12-HETE in UVR-exposed skin between the active group (43.1 ± 4.6 pg/μL) and the placebo group (38.9 ± 4.9 pg/μL; *P* = 0.45; Figure 5B). Results were comparable after multiple imputation and per-protocol analyses. Results for all eicosanoid species currently assayed in blister fluid are presented in **Supplemental Table 1**. There was no statistically significant impact of GTC supplementation on basal or UVR-induced production of these eicosanoids.

## DISCUSSION

Green tea is suspected to have benefits to health spanning a wide range of inflammatory and immune-mediated disorders (36), in addition to chemopreventive properties. However, limited information is available from human studies, with most data arising from experimental study of the most abundant GTC: EGCG. Skin is exposed to and requires protection from environmental UVR and itself provides an accessible organ for direct assessment of impact of in vivo supplementation in humans, facilitating noninvasive measurements, the administration of external challenges, and comparative ease and safety of tissue sampling. Our 3-mo supplementation trial was performed under controlled conditions, evaluating the impact of GTC ingestion on skin inflammatory status under basal conditions and after a reproducible acute UVR challenge to assess GTCs’ photoprotective potential.

In this double-blind randomized controlled trial, we found that oral GTC supplementation taken with low-dose vitamin C did not have a statistically significantly effect on the skin basal inflammatory status or the response to acute proinflammatory UVR challenge, including clinical and histologic sunburn outcomes, or their biochemical mediation through major proinflammatory eicosanoids produced via the cyclooxygenase and lipoxygenase pathways. Thus, compared with placebo, oral supplementation with GTCs for 12 wk did not reduce UVR-induced inflammation in terms of the clinical erythema response, assessed both visually as the sunburn threshold (MED) and quantitatively across the UVR erythema dose-response (AUC for UVR dose vs. erythema index). Similarly, consumption of GTCs did not have a statistically significantly effect on infiltration of neutrophils or T cells after UVR challenge. Finally, GTCs did not have a statistically significantly effect on baseline or UVR-induced concentrations of PGE_2_ and 12-HETE, which have roles in the mediation of sunburn vasodilatation and chemoattraction, respectively (9). These data were obtained from volunteers demonstrated to be compliant with their supplementation.

There are several mechanisms by which GTCs might potentially suppress the sunburn response, including downregulation of cyclooxygenase and/or lipoxygenase expression as demonstrated in various cell models (18, 19, 37, 38). Other mechanisms include reduced production of proinflammatory and chemoattractant cytokines such as IL-1α and TNF-α, inhibition of T-cell proliferation (36), and upregulation of antioxidant enzyme concentrations (39, 40). These activities are supported by studies in rodent models, where green tea constituents modulated nuclear transcription factor κB signaling (24), attenuated UVR-induced cyclooxygenase activity (41), and reduced neutrophil migration to sites of inflammation (22, 23). Topical application of GTCs was also reported to reduce UVB-induced production of nitric oxide and hydrogen peroxide in a mouse model (42). In addition to the above, cyclooxygenase and lipoxygenase are molecular targets for other related polyphenols, with reports of reduced eicosanoid production by lipoxygenase isoforms, including 12-lipoxygenase, in vitro (43, 44).

The above mechanistic studies and our pilot human study (27) indicated examination for the impact of oral GTC supplementation on the lipoxygenase metabolite and leukocyte chemoattractant 12-HETE, alongside cutaneous leukocyte infiltration. We did not observe a statistically significant difference in neutrophil numbers between supplementation groups or any difference in 12-HETE concentrations. Although topical application of GTCs before UVR exposure was reported to reduce UVB-induced leukocyte infiltration in animal models (19, 42) and in humans (25), the current study is the first to report the impact of orally consumed GTCs on leukocyte infiltration and production of cyclooxygenase/lipoxygenase metabolites in humans, as well as after a solar-simulated radiation challenge.

The UVR erythema response findings in our double-blind randomized controlled trial are in contrast to those in our previous open, uncontrolled study indicating GTCs might protect against UVR-induced erythema. This was a modest effect, evident as a reduction of the measured erythema index at the highest dose of UVR tested (27), and followed supplementation with 540 mg GTC/d and 50 mg vitamin C/d (i.e., half the amount of the current study). It is important to acknowledge the limitations of open, uncontrolled studies, particularly the inability to allow for changes in outcome measures during the course of supplementation that are unrelated to consumption of the supplement. There is also the possibility of a nonlinear dose-response effect, because higher concentrations of nutritional supplementation are sometimes reported to be less effective (e.g., 200 mg vitamin E/d was more effective than 800 mg/d in enhancing T-cell responses in the elderly) (45). However, the other published human study exploring the impact of orally consumed GTCs on sunburn, which examined for and found a suppressive effect on the visual erythema threshold (i.e., after a low-dose UVR challenge) used a higher dose of GTCs (26). The participants were instructed to consume 1 L/d of a green tea beverage (1402 mg GTC with 119 mg vitamin C daily); this also contained a higher proportion of EGCG (daily: 100 mg epicatechin, 980 mg EGCG, and 238 mg epicatechin gallate) than our supplement (daily: 75 mg epicatechin, 436 mg EGCG, and 156 mg epicatechin gallate), and this may be the most biologically active GTC (46). Other differences are that in our pilot study, the subject group comprised nearly all females, raising the possibility of a sex-specific effect; the BMI of the group was lower; and the challenges posed to rigorous blinding in a beverage study.

Strengths of this study include the double-blind randomized controlled design, a statistically justified sample size, and biochemical confirmation of subject compliance. We took an oral consumption approach rather than topical application, thus eliminating the possibility of a contribution of local protection from the UVR challenge by a sunscreen effect (47) and with greater relevance to human nutrition. Objective measurement of sunburn erythema was made across the UVR dose series, enabling characterization of responses to threshold, moderate- and high-dose solar-simulated UVR, and the impact of GTCs on these. In view of ethical considerations and the range of samples taken in this study, there was not scope to take further skin tissue for assessment of target organ (i.e., skin) bioavailability of GTCs. Potentially, interindividual variability in the types of catechins and metabolites in skin postsupplementation, as demonstrated in our pilot study (27), may influence outcomes.

We conclude that in a compliant mixed-sex study population, the daily consumption of 1080 mg encapsulated GTC, equivalent to 5 cups of green tea, taken with 100 mg vitamin C, does not significantly affect the acute UVR-induced erythema (sunburn) response, dermal leukocytic infiltration, or the cutaneous production of proinflammatory cyclooxygenase and lipoxygenase metabolites. Future studies might examine EGCG dose effect, discuss the impact on other chemokines, and explore the skin content of GTCs and their metabolites.

## Footnotes

7 Abbreviations used: EGCG, (−)-epigallocatechin-3-*O*-gallate; GTC, green tea catechin; HETE, hydroxyeicosatetraenoic acid; hpf, high-power field; MED, minimal erythema dose; PG, prostaglandin; UVR, ultraviolet radiation.

## References

[1] Ferguson J European guidelines (COLIPA) for evaluation of sun protection factors. In: Lowe NJ, Shaath NA, Pathak MA, editors. Sunscreens: development, evaluation and regulatory aspects. New York: Marcel Dekker; 1997; p. 513–25.

[2] Azurdia RM, Pagliaro JA, Diffey BL, Rhodes LE Sunscreen application by photosensitive patients is inadequate for protection. Br J Dermatol 1999;140:255–8. 10233218 10.1046/j.1365-2133.1999.02658.x

[3] Jansen R, Wang SQ, Burnett M, Osterwalder U, Lim HW Photoprotection: part I. Photoprotection by naturally occurring, physical, and systemic agents. J Am Acad Dermatol 2013;69:853.e1–12. 24238179 10.1016/j.jaad.2013.08.021

[4] Hawk JL, Murphy GM, Holden CA The presence of neutrophils in human cutaneous ultraviolet-B inflammation. Br J Dermatol 1988;118:27–30. 3342174 10.1111/j.1365-2133.1988.tb01746.x

[5] Strickland I, Rhodes LE, Flanagan BF, Friedmann PS TNF-α and IL-8 are upregulated in the epidermis of normal human skin after UVB exposure: correlation with neutrophil accumulation and E-selectin expression. J Invest Dermatol 1997;108:763–8. 9129230 10.1111/1523-1747.ep12292156

[6] Chen X, Gresham A, Morrison A, Pentland AP Oxidative stress mediates synthesis of cytosolic phospholipase A2 after UVB injury. Biochim Biophys Acta 1996;1299:23–33. 8555249 10.1016/0005-2760(95)00166-2

[7] Gresham A, Masferrer J, Chen X, Leal-Khouri S, Pentland AP Increased synthesis of high-molecular-weight cPLA_2_ mediates early UV-induced PGE_2_ in human skin. Am J Physiol 1996;270:C1037–50. 8928731 10.1152/ajpcell.1996.270.4.C1037

[8] Black AK, Fincham N, Greaves MW, Hensby CN Time course changes in levels of arachidonic acid and prostaglandins D_2_, E_2_, F_2α_ in human skin following ultraviolet B irradiation. Br J Clin Pharmacol 1980;10:453–7. 7437257 10.1111/j.1365-2125.1980.tb01788.x PMC1430161

[9] Rhodes LE, Gledhill K, Masoodi M, Haylett AK, Brownrigg M, Thody AJ, Tobin DJ, Nicolaou A The sunburn response in human skin is characterized by sequential eicosanoid profiles that may mediate its early and late phases. FASEB J 2009;23:3947–56. 19584301 10.1096/fj.09-136077 PMC2791058

[10] Goetzl EJ, Brash AR, Tauber AI, Oates JA, Hubbard WC Modulation of human neutrophil function by monohydroxy-eicosatetraenoic acids. Immunology 1980;39:491–501. 6247265 PMC1458022

[11] Dowd PM, Kobza Black A, Woollard PM, Camp RD, Greaves MW Cutaneous responses to 12-hydroxy-5,8,10,14-eicosatetraenoic acid (12-HETE). J Invest Dermatol 1985;84:537–41. 3998504 10.1111/1523-1747.ep12273537

[12] Lorenz M Cellular targets for the beneficial actions of tea polyphenols. Am J Clin Nutr 2013;98:1642S–50S. 24172299 10.3945/ajcn.113.058230

[13] Baliga MS, Katiyar SK Chemoprevention of photocarcinogenesis by selected dietary botanicals. Photochem Photobiol Sci 2006;5:243–53. 16465310 10.1039/b505311k

[14] Lambert JD, Elias RJ The antioxidant and pro-oxidant activities of green tea polyphenols: a role in cancer prevention. Arch Biochem Biophys 2010;501:65–72. 20558130 10.1016/j.abb.2010.06.013 PMC2946098

[15] Hooper L, Kroon PA, Rimm EB, Cohn JS, Harvey I, Le Cornu KA, Ryder JJ, Hall WL, Cassidy A Flavonoids, flavonoid-rich foods, and cardiovascular risk: a meta-analysis of randomized controlled trials. Am J Clin Nutr 2008;88:38–50. 18614722 10.1093/ajcn/88.1.38

[16] Lee KW, Kundu JK, Kim SO, Chun KS, Lee HJ, Surh YJ Cocoa polyphenols inhibit phorbol ester-induced superoxide anion formation in cultured HL-60 cells and expression of cyclooxygenase-2 and activation of NF-kappaB and MAPKs in mouse skin in vivo. J Nutr 2006;136:1150–5. 16614396 10.1093/jn/136.5.1150

[17] Leifert WR, Abeywardena MY Grape seed and red wine polyphenol extracts inhibit cellular cholesterol uptake, cell proliferation, and 5-lipoxygenase activity. Nutr Res 2008;28:842–50. 19083497 10.1016/j.nutres.2008.09.001

[18] Hong J, Smith TJ, Ho CT, August DA, Yang CS Effects of purified green and black tea polyphenols on cyclooxygenase and lipoxygenase-dependent metabolism of arachidonic acid in human colon mucosa and colon tumour tissues. Biochem Pharmacol 2001;62:1175–83. 11705450 10.1016/s0006-2952(01)00767-5

[19] Meeran SM, Akhtar S, Katiyar SK Inhibition of UVB-induced skin tumor development by drinking green tea polyphenols is mediated through DNA repair and subsequent inhibition of inflammation. J Invest Dermatol 2009;129:1258–70. 19020550 10.1038/jid.2008.354 PMC2669839

[20] Peng G, Dixon DA, Muga SJ, Smith TJ, Wargovich MJ Green tea polyphenol (−)-epigallocatechin-3-gallate inhibits cyclooxygenase-2 expression in colon carcinogenesis. Mol Carcinog 2006;45:309–19. 16508969 10.1002/mc.20166

[21] Afaq F, Adhami VM, Ahmad N, Mukhtar H Inhibition of ultraviolet B–mediated activation of nuclear factor kappaB in normal human epidermal keratinocytes by green tea constituent (−)-epigallocatechin-3-gallate. Oncogene 2003;22:1035–44. 12592390 10.1038/sj.onc.1206206

[22] Donà M, Dell’Aica I, Calabrese F, Benelli R, Morini M, Albini A, Garbisa S Neutrophil restraint by green tea: inhibition of inflammation, associated angiogenesis, and pulmonary fibrosis. J Immunol 2003;170:4335–41. 12682270 10.4049/jimmunol.170.8.4335

[23] Takano K, Nakaima K, Nitta M, Shibata F, Nakagawa H Inhibitory effect of (−)-epigallocatechin 3-gallate, a polyphenol of green tea, on neutrophil chemotaxis in vitro and in vivo. J Agric Food Chem 2004;52:4571–6. 15237969 10.1021/jf0355194

[24] Afaq F, Ahmad N, Mukhtar H Suppression of UVB-induced phosphorylation of mitogen-activated protein kinases and nuclear factor kappa B by green tea polyphenol in SKH-1 hairless mice. Oncogene 2003;22:9254–64. 14681684 10.1038/sj.onc.1207035

[25] Katiyar SK, Matsui MS, Elmets CA, Mukhtar H Polyphenolic antioxidant (−)-epigallocatechin-3-gallate from green tea reduces UVB-induced inflammatory responses and infiltration of leukocytes in human skin. Photochem Photobiol 1999;69:148–53. 10048310

[26] Heinrich U, Moore CE, De Spirt S, Tronnier H, Stahl W Green tea polyphenols provide photoprotection, increase microcirculation, and modulate skin properties of women. J Nutr 2011;141:1202–8. 21525260 10.3945/jn.110.136465

[27] Rhodes LE, Darby G, Massey KA, Clarke KA, Dew TP, Farrar MD, Bennett S, Watson REB, Williamson G, Nicolaou A Oral green tea catechin metabolites are incorporated into human skin and protect against UVR-induced cutaneous inflammation in association with reduced production of pro-inflammatory eicosanoid 12-HETE. Br J Nutr 2013;110:891–900. 23351338 10.1017/S0007114512006071

[28] Chen ZY, Zhu QY, Wong YF, Zhang ZS, Chung HY Stabilizing effect of ascorbic acid on green tea catechins. J Agric Food Chem 1998;46:2512–6.

[29] McArdle F, Rhodes LE, Parslew R, Jack CIA, Friedmann PS, Jackson MJ UVR-induced oxidative stress in human skin in vivo: effects of vitamin C supplementation. Free Radic Biol Med 2002;33:1355–62. 12419467 10.1016/s0891-5849(02)01042-0

[30] Masoodi M, Nicolaou A Lipidomic analysis of twenty-seven prostanoids and isoprostanes by liquid chromatography/electrospray tandem mass spectrometry. Rapid Commun Mass Spectrom 2006;20:3023–9. 16986207 10.1002/rcm.2697 PMC1805459

[31] Masoodi M, Mir AA, Petasis NA, Serhan CN, Nicolaou A Simultaneous lipidomic analysis of three families of bioactive lipid mediators leukotrienes, resolvins, protectins and related hydroxy-fatty acids by liquid chromatography/electrospray tandem mass spectrometry. Rapid Commun Mass Spectrom 2008;22:75–83. 18059001 10.1002/rcm.3331 PMC2542421

[32] Massey KA, Nicolaou A Lipidomics of oxidized polyunsaturated fatty acids. Free Radic Biol Med 2013;59:45–55. 22940496 10.1016/j.freeradbiomed.2012.08.565 PMC3991857

[33] Clarke KA, Dew TP, Watson REB, Farrar MD, Bennett S, Nicolaou A, Rhodes LE, Williamson G High performance liquid chromatography tandem mass spectrometry dual extraction method for identification of green tea catechin metabolites excreted in human urine. J Chromatogr B Analyt Technol Biomed Life Sci 2014;972:29–37. 10.1016/j.jchromb.2014.09.035 25306116

[34] Swindells K, Rhodes LE Influence of oral antioxidants on UVR-induced skin damage in humans. Photodermatol Photoimmunol Photomed 2004;20:297–304. 15533237 10.1111/j.1600-0781.2004.00121.x

[35] Heinrich U, Neukam K, Tronnier H, Sies H, Stahl W Long-term ingestion of high flavanol cocoa provides photoprotection against UV-induced erythema and improves skin condition in women. J Nutr 2006;136:1565–9. 16702322 10.1093/jn/136.6.1565

[36] Pae M, Wu D Immunomodulating effects of epigallocatechin-3-gallate from green tea: mechanisms and applications. Food Funct 2013;4:1287–303. 23835657 10.1039/c3fo60076a

[37] Hussain T, Gupta S, Adhami VM, Mukhtar H Green tea constituent epigallocatechin-3-gallate selectively inhibits COX-2 without affecting COX-1 expression in human prostate carcinoma cells. Int J Cancer 2005;113:660–9. 15455372 10.1002/ijc.20629

[38] Koeberle A, Bauer J, Verhoff M, Hoffmann M, Northoff H, Werz O Green tea epigallocatechin-3-gallate inhibits microsomal prostaglandin E(2) synthase-1. Biochem Biophys Res Commun 2009;388:350–4. 19665000 10.1016/j.bbrc.2009.08.005

[39] Colon M, Nerin C Role of catechins in the antioxidant capacity of an active film containing green tea, green coffee, and grapefruit extracts. J Agric Food Chem 2012;60:9842–9. 22973940 10.1021/jf302477y

[40] Meng Q, Velalar CN, Ruan R Effects of epigallocatechin-3-gallate on mitochondrial integrity and antioxidative enzyme activity in the aging process of human fibroblast. Free Radic Biol Med 2008;44:1032–41. 18206666 10.1016/j.freeradbiomed.2007.11.023

[41] Agarwal R, Katiyar SK, Khan SG, Mukhtar H Protection against ultraviolet B radiation–induced effects in the skin of SKH-1 hairless mice by a polyphenolic fraction isolated from green tea. Photochem Photobiol 1993;58:695–700. 8284325 10.1111/j.1751-1097.1993.tb04954.x

[42] Katiyar SK, Mukhtar H Green tea polyphenol (−)-epigallocatechin-3-gallate treatment to mouse skin prevents UVB induced infiltration of leukocytes, depletion of antigen presenting cells and oxidative stress. J Leukoc Biol 2001;69:719–26. 11358979

[43] Sies H, Schewe T, Heiss C, Kelm M Cocoa polyphenols and inflammatory mediators. Am J Clin Nutr 2005;81:304S–12S. 15640495 10.1093/ajcn/81.1.304S

[44] Schewe T, Sadik C, Klotz LO, Yoshimoto T, Kühn H, Sies H Polyphenols of cocoa: inhibition of mammalian 15-lipoxygenase. Biol Chem 2001;382:1687–96. 11843182 10.1515/BC.2001.204

[45] Meydani SN, Meydani M, Blumberg JB, Leka LS, Siber G, Loszewski R, Thompson C, Pedrosa MC, Diamond RD, Stollar BD Vitamin E supplementation and in vivo immune response in healthy elderly subjects: a randomized controlled trial. JAMA 1997;277:1380–6. 9134944 10.1001/jama.1997.03540410058031

[46] Elmets CA, Singh D, Tubesing K, Matsui M, Katiyar S, Mukhtar H Cutaneous photoprotection from ultraviolet injury by green tea polyphenols. J Am Acad Dermatol 2001;44:425–32. 11209110 10.1067/mjd.2001.112919

[47] Lu LY, Ou N, Lu Q-B Antioxidant induces DNA damage, cell death and mutagenicity in human lung and skin normal cells. Sci Rep 2013;3:3169. 24201298 10.1038/srep03169 PMC3821017

